# Assessing the “Optimism–Knowledge Gap”: An Exploratory Study of AI Awareness, Application, and Educational Needs Among a Sample of Italian Clinicians

**DOI:** 10.3390/healthcare14070847

**Published:** 2026-03-26

**Authors:** Alessandro Perrella, Pierpaolo di Micco, Ugo Trama, Pierino di Silverio, Ada Maffettone, Gaetano Piccinocchi, Francesca Futura Bernardi

**Affiliations:** 1 Emerging Infectious Disease and High Contagiousness Unit, P.O. D. Cotugno-AORN Ospedali dei Colli, 80131 Naples, Italy; pierino.disilverio@ospedalideicolli.it; 2 Regional Observatory for Infectious Disease, 80100 Naples, Italy; 3 AFO Medicina, P.O. Santa Maria delle Grazie, 80076 Pozzuoli, Italy; pdimicco@libero.it; 4 ASL Napoli 2 Nord, 80076 Naples, Italy; 5 Coordination of the Regional Health System, General Directorate for Health Protection, 80143 Naples, Italy; ugo.trama@regione.campania.it (U.T.); francescafutura.bernardi@regione.campania.it (F.F.B.); 6 Internal Medicine Unit, Ospeale San Luca, 84078 Vallo della Lucania, Italy; adamaff@hotmail.com; 7 ASL Salerno, 84124 Salerno, Italy; 8 Coordination SIMG, Campania Region, 50142 Florence, Italy; piccinocchi.gaetano@simg.it

**Keywords:** artificial intelligence, clinical competence, human-in-the-loop, general practice, digital health literacy

## Abstract

**Background:** Artificial intelligence (AI) is poised to fundamentally reshape healthcare delivery, offering unprecedented advancements in diagnostics, treatment personalization, and operational efficiency. However, a growing body of international research reveals a critical “optimism–knowledge gap”: healthcare professionals are enthusiastic about AI’s potential but possess limited technical knowledge and practical experience. This gap compromises the safe and effective implementation of AI tools. The Italian healthcare context presents a unique and amplifying challenge, as it is defined by the stringent “human-in-the-loop” oversight mandated by the Garante per la protezione dei dati personali (Italy’s Data Protection Authority). This legal framework makes clinician competence not just a goal, but a prerequisite for regulatory compliance. **Objective:** This study aimed to provide an exploratory quantitative assessment of AI awareness, practical application, and understanding of its limitations among a sample of clinicians in Italy. It specifically sought to compare the preparedness of hospital-based clinicians and general practitioners (GPs) and to identify the workforce’s perceived educational needs within this unique legal environment. **Methods:** A descriptive, cross-sectional survey was conducted from February to August 2025. Using a non-probability convenience sampling method via professional networks, the survey yielded 362 total responses. Data were analyzed descriptively and inferentially using Chi-square ($\chi^{2}$) tests to compare cohort responses on familiarity, practical exposure, knowledge of limitations, and interest in further training. **Results:** A universal and high demand for education was found, with 89.9% of all respondents being “Moderately” or “Very” interested in learning more about AI. This optimism coexists with dangerously low practical exposure. The gap was most profound among GPs, 44.1% of whom have “Never” used an AI tool—a rate significantly higher than hospital clinicians (34.9%; $\chi^{2}=3.14$, *p* = 0.045). Furthermore, 32.6% of GPs admitted that they “understand some benefits but not the limitations.” **Conclusions:** Italian clinicians mirror the global optimism–knowledge gap. These findings underscore the urgent need for structured, continuous education in AI literacy to address ethical and regulatory imperatives within the Italian healthcare system.

## 1. Introduction

Artificial intelligence (AI), particularly in its forms of machine learning (ML) and deep learning (DL), represents one of the most significant technological shifts in modern medicine [1]. Its potential is transformative, promising to augment human capability at every level of care, from predictive analytics managing patient flow to sophisticated imaging algorithms detecting malignancies with superhuman accuracy [2]. However, the integration of AI is not merely a technical upgrade; it is a fundamental restructuring of the clinical relationship.

While the literature often focuses on efficiency, a more nuanced challenge exists: the tension between algorithmic precision and the messy, interpersonal reality of patient care. AI offers the promise of relieving clinicians from administrative burdens, theoretically freeing them to focus on “human” tasks [3]. However, it also introduces a “Transparency Paradox”: providing complex “explainability” data to clinicians without the foundational literacy to interpret it may increase cognitive load rather than clarity [4,5]. The “black box” nature of many AI systems can create a barrier of opacity, making it difficult for clinicians to explain diagnoses to patients, thereby threatening the trust that is foundational to the therapeutic alliance. If transparency is mishandled, it risks overwhelming patients with probabilistic data they cannot interpret, turning a tool of empowerment into a source of confusion.

As AI tools move from research labs to frontline deployment, a consistent trend has emerged globally: the “optimism–knowledge gap.” Studies from diverse healthcare systems have been pivotal in defining this phenomenon. For instance, recent research in South Asia found that while over 80% of healthcare professionals believed AI could improve accuracy, fewer than 20% felt confident in their own technical knowledge [6]. Similarly, data from the Middle East highlights that high optimism often coexists with significant fears regarding job displacement and a lack of empathy in AI systems [4]. This gap creates a dangerous “blind spot,” rendering clinicians vulnerable to “automation bias”—the tendency to favor automated suggestions even when they are incorrect [7]. This disconnect is fueled by a systemic failure in medical education, which has historically treated digital literacy as a niche skill rather than a core competency [5,7].

### The Unique Italian Context: The “Human-in-the-Loop” Mandate

This study situates the “optimism–knowledge gap” within the specific and highly regulated context of the Italian National Healthcare System. Unlike jurisdictions that prioritize rapid innovation, Italy is governed by one of the world’s most robust data protection frameworks, overseen by the Garante per la protezione dei dati personali [8]. The Garante has classified healthcare AI as “high-risk” [9] and established a strict “human-in-the-loop” mandate. Under Italian law (e.g., L. 132/2025) and the Garante’s “decalogo,” the clinician is legally required to “control, confirm, or refute” the AI’s suggestion [10,11]. Legally adequate “supervision” requires the clinician to possess the specific competence to understand why a model might fail due to dataset bias or algorithmic drift. If a clinician lacks this competence, they become a “rubber stamp,” providing the illusion of human oversight without the substance, potentially exposing themselves and their institutions to significant liability.

## 2. Materials and Methods

### 2.1. Study Design and Population

A descriptive, cross-sectional survey design was employed to capture a comprehensive snapshot of AI awareness among practicing clinicians in Italy. The study population comprised licensed healthcare professionals, including physicians, nurses, and allied health roles, ensuring a multidisciplinary perspective. To enable a granular analysis of workforce preparedness and to test the hypothesis of a “digital divide” between care settings, the sample (n = 362) was categorized into three distinct cohorts. This segmentation allows for the comparison of resource-rich environments (hospitals) versus community-based settings (general practice) (Table 1). Specialists in radiology or other medical fields like laboratory medicine, who may have had previous experience with AI-based approaches in their clinical activities, were excluded to avoid bias from participants already skilled in AI. The survey instrument was developed based on a capability- and function-oriented review of AI in healthcare to ensure that items reflected current technological realities. The structure and item categorization were specifically adapted from the validated methodology established in our previous work (Perrella et al., 2024 [12]), which utilized a similar framework for assessing clinician readiness and ethical AI integration. To ensure construct validity, the questionnaire underwent face validity assessment by a multidisciplinary panel of clinical experts. A pilot test was conducted with a small group of clinicians (n = 10) to refine item wording and resolve ambiguities before the final electronic distribution

### 2.2. Sampling and Data Collection Flow

**Figure 1:**
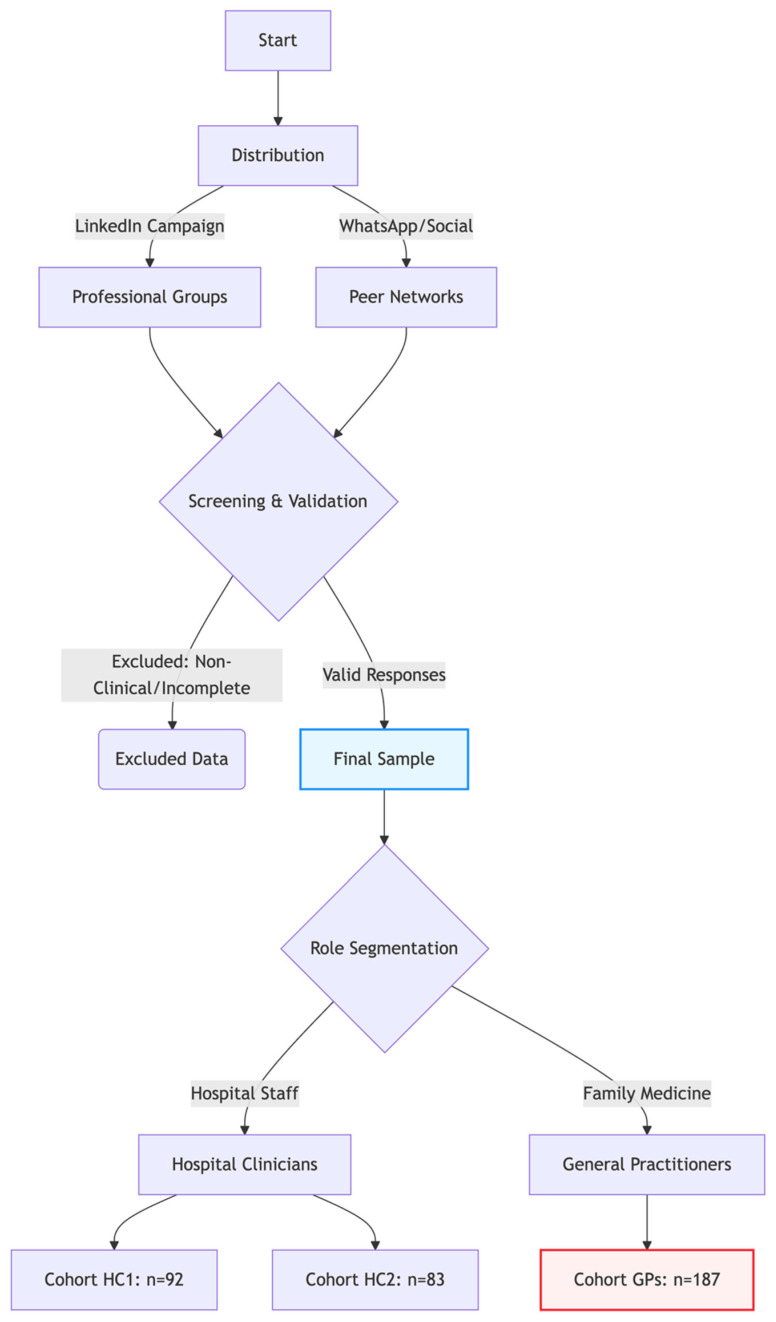
Study recruitment and cohort allocation flow chart.

The survey was conducted from February to August 2025. A non-probability convenience sampling method was utilized, recruiting participants via professional networks (LinkedIn (https://in.linkedin.com/)/WhatsApp (https://web.whatsapp.com/)). We acknowledge that this strategy inherently selects for a “digitally active” segment of the workforce, which should be considered when interpreting generalizability. The recruitment process and allocation into cohorts are visualized in Figure 1.

### 2.3. Data Analysis

Data were exported and aggregated for descriptive analysis. Frequencies and percentages were calculated to summarize responses. Statistical analyses were performed using jamovi (version 2.3) and Python (3.12.12)-based scripts for advanced data processing. Frequencies and percentages summarized the responses. To establish the significance of observed disparities, Chi-square ($\chi^{2}$) tests were performed using jamovi (version 2.3). Comparative analysis focused on the gap between GPs and the combined hospital clinicians (HC1 + HC2) regarding familiarity and practical application.

## 3. Results

**Figure 2:**
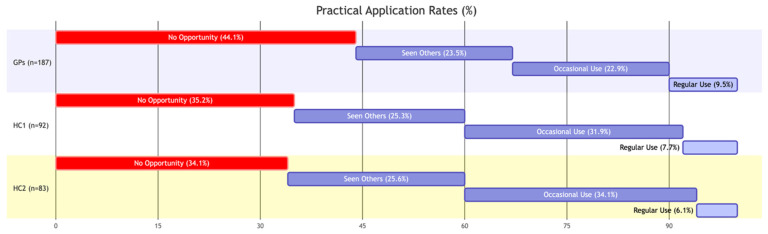
Practical application of AI tools by clinician group (Q8). Question: “Have you ever used any AI-powered tools or software in your work?” (Note: Red bars indicate “No Opportunity,” blue bars represents grade of knowledge, showcasing the profound lack of access in primary care). See more detail in Supplementary Table S1.

The analysis reveals a healthcare landscape characterized by high theoretical interest but low practical capability. The data confirms the existence of a “two-speed” digitization process in Italy. A stark disparity emerged regarding practical exposure. While hospital clinicians operate in environments where AI might be integrated into PACS or EHR systems, GPs often lack access to these tools. As illustrated in Figure 2, the rate of non-adoption among GPs is significantly higher than in hospital settings. Specifically, 44.1% of GPs reported “No opportunity” to use AI, compared to 34.9% of hospital clinicians ($\chi^{2}=3.14$, *p* = 0.045). Only 9.5% of GPs use these tools regularly, suggesting that AI remains a theoretical concept in primary care.

**Figure 3:**
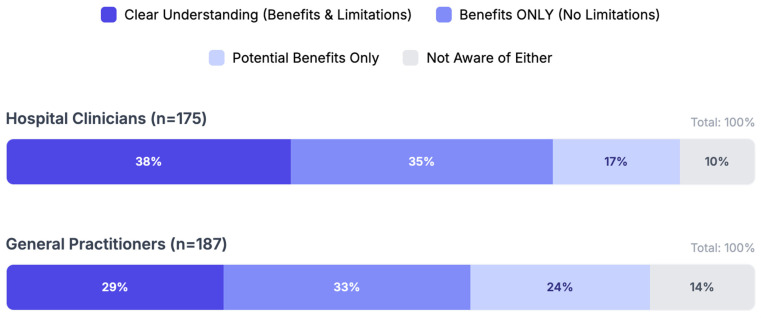
Comparative assessment of AI understanding between general practitioners (GPs) and hospital clinicians. The stacked bar chart illustrates the distribution of self-reported knowledge levels regarding AI capabilities and constraints. Categories are defined as Figure 4.

Perhaps the most concerning finding is the qualitative nature of the knowledge gap. The survey asked participants to rate their understanding of both the benefits and limitations of AI. The prevalence of “uninformed optimism” appears notably high among GPs; 32.6% admitted they understand benefits but not limitations. This suggests an exposure to marketing narratives without corresponding education in risks such as algorithmic drift or hallucinations (Figure 3). In contrast, hospital clinicians demonstrated a slightly more balanced view, though a significant portion still lacked comprehensive understanding.

**Figure 4:**
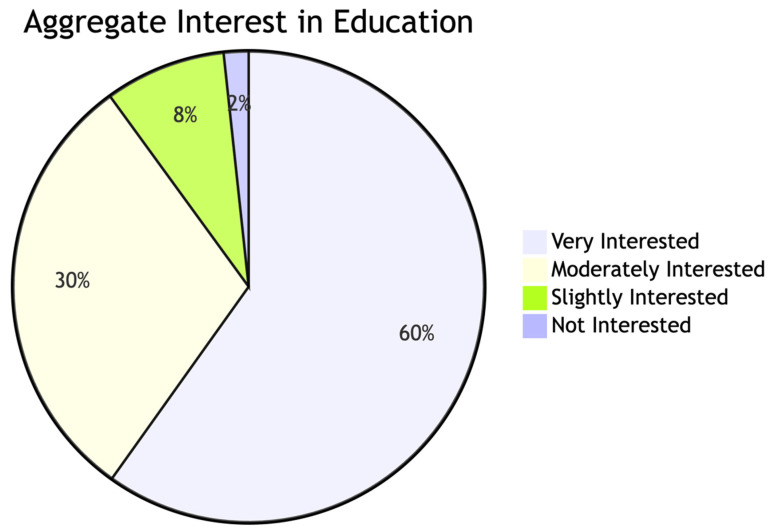
Aggregate interest in AI education (n = 362). Question: “How would you rate your interest in learning more about AI?”. See more detail in Supplementary Table S3.

### The Universal Demand for Education

Despite the gaps in usage and knowledge, the workforce is not resistant. On the contrary, there is a nearly universal demand for training. As shown in Figure 4, 89.9% of the total sample expressed moderate to high interest in learning more. This indicates that the “gap” is not one of motivation, but of opportunity and institutional support.

## 4. Discussion

The results highlight a critical tension that extends beyond simple “tech literacy.” While the Garante mandates transparency as a prerequisite for safety [10], our results suggest that for an untrained workforce, transparency paradoxically risks increasing cognitive load [13].

The 32.6% of GPs who understand benefits but not limitations appear particularly vulnerable to automation bias [14]. If transparency mechanisms are too dense to be useful in a 15 min consultation, clinicians may default to “Blind Trust,” accepting AI outputs without verification to save time. This turns the “human-in-the-loop” into a “rubber stamp,” undermining the intent of Law 132/2025.

Furthermore, the requirement for human supervision imposes an “emotional labor.” The clinician must manage their own uncertainty regarding the “black box” while projecting confidence to the patient. This “bridging” requires deep AI literacy; without it, the clinician is left to manage the anxiety of potential liability alone.

Indeed, they may be overwhelmed by the “black box” nature of the tool, leading to two equally dangerous outcomes: (1) Blind Trust, where the clinician ignores the complex explanations and simply accepts the AI’s output to save time; or (2) Paralysis/Rejection, where the clinician avoids the use of the tool entirely because the transparency mechanisms are too dense to be useful in a 15 min consultation.

True transparency, therefore, requires more than just disclosing algorithms; it requires a workforce capable of receiving that disclosure without being paralyzed by it. The high levels of interest (89.9%) coupled with the low levels of usage (44.1% non-use among GPs) point to a complex psychological relationship with the technology. Clinicians appear to view AI as a potential ally that is currently inaccessible. However, the “optimism–knowledge gap” suggests that this alliance is fragile. The enthusiasm is likely predicated on the idea of AI as a workload reducer. If the actual implementation of AI—mandated by the Garante to include rigorous, active human supervision—turns out to increase the workload (by requiring constant verification of outputs), the “ally” may quickly be perceived as an “alien” intruder. The “human-in-the-loop” model turns the clinician into a supervisor of a machine they do not fully understand, a role shift that many did not sign up for and for which they feel unprepared. The Garante’s requirement for human supervision imposes not just a legal burden, but an emotional one. “Human-in-the-loop” is often discussed as a safety mechanism, but for the clinician, it is a form of emotional labor. Finally, the knowledge gap implies clinicians may be unaware of dataset bias. Italy’s demographics are changing, and if models are trained on historical, homogeneous populations, they may perform poorly on minority groups. A clinician unaware of limitations will not ask if an AI is accurate for a specific patient demographic, potentially making the lack of education a vector for systemic discrimination. Therefore, the lack of education identified in this study is not just a technical deficit; it is a vector for potential systemic discrimination. An educated workforce is the only firewall against algorithmic bias [12,13,14].

We acknowledge several limitations: the convenience sampling method overrepresents tech-engaged clinicians, and the cross-sectional design prevents causal inference. Future research should utilize randomized sampling and objective competency measures.

## 5. Conclusions

This study confirms that Italian clinicians are caught in a classic “optimism–knowledge gap” [12]. While enthusiasm is high, the “human-in-the-loop” mandate enshrined in Italian law is currently unsupported by the reality of workforce competence. The disparity identified between hospital clinicians and GPs appears significant, threatening a two-tiered health system. Furthermore, the analysis suggests that without addressing the psychological and emotional dimensions of AI adoption, specifically the cognitive load of supervision and the anxiety of liability, legal mandates alone will not ensure safety [13,14]. In our opinion, to advance a realistic AI-based professional approach, we should now focus on

1. Curriculum Reform: Medical education must move beyond “digital literacy” to “AI competency,” specifically teaching the limitations and failure modes of AI to inoculate against automation bias.
2. Differentiated Training: Specific programs must be designed for GPs that account for their decentralized work environment and lack of institutional IT support.
3. Supportive Policy: The Garante and health institutions should recognize that “human supervision” is a form of labor that requires time, resources, and protection.

Fostering deep AI literacy is not just about teaching code; it is about empowering the “human” in the loop to remain the true, competent guardian of patient care [12,13,14].

## Figures and Tables

**Table 1:** Definition of study cohorts.

Cohort ID	Description	Sample Size (n)	Professional Role Breakdown	Median Clinical Experience (IQR)	Primary Setting
HC1	Hospital Clinicians (G1)	92	Physicians: 65%; Nurses: 28%; Allied: 7%	14 yrs (8–22)	General Hospital
HC2	Hospital Clinicians (G2)	83	Physicians: 72%; Nurses: 20%; Allied: 8%	12 yrs (6–20)	Specialist/Academic
GPs	General Practitioners	187	Physicians: 100%	18 yrs (10–28)	Primary Care
Total	All Participants	362	Physicians: 85%; Nurses: 12%; Allied: 3%	15 yrs (8–25)	-

## Data Availability

Data is unavailable due to privacy and ethical restrictions.

## Supplementary Materials

The following supporting information can be downloaded at: https://www.mdpi.com/article/10.3390/healthcare14070847/s1, Table S1: Practical Application Frequency (Q8). Table S2: Understanding of Benefits vs. Limitations (Q7). Table S3: Interest in AI Education (Aggregate).
