# Supplementary material for: Assessing the “Optimism–Knowledge Gap”: An Exploratory Study of AI Awareness, Application, and Educational Needs Among a Sample of Italian Clinicians
Source: Healthcare (Basel). 2026 Mar 26;14(7):847. doi: 10.3390/healthcare14070847 (PMC13073766; doi:10.3390/healthcare14070847)
Supplement: Supplementary file 1 [file healthcare-14-00847-s001.zip › healthcare-4204452-supplementary.pdf]

Supplementary Material: Aggregated Survey Data

Article: Assessing the “Optimism-Knowledge Gap”: An Exploratory Study of AI Awareness, Application and Educational Needs Among a Sample of Italian Clinicians

Table S1: Practical Application Frequency (Q8)

Question: "Have you ever used any AI-powered tools or software in your work?"

Response Category	GPs (n=187)	Hospital Clinicians (n=175)
No Opportunity (Never)	82 (44.1%)	61 (34.9%)
Seen Others Use It	44 (23.5%)	45 (25.7%)
Occasional Use	43 (22.9%)	56 (32.0%)
Regular Use	18 (9.5%)	13 (7.4%)

Table S2: Understanding of Benefits vs. Limitations (Q7)

Question: "To what extent do you understand the potential benefits and limitations of AI?"

Response Category	GPs (n=187)	Hospital Clinicians (n=175)
Clear Understanding (Both)	54 (29%)	67 (38%)
Benefits ONLY (No Limitations)	61 (33%)	61 (35%)
Potential Benefits Only	45 (24%)	30 (17%)
Not Aware of Either	27 (15%)	17 (11%)

Table S3: Interest in AI Education (Aggregate)

Question: "How would you rate your interest in learning more about AI?" (N=362)

Interest Level	Frequency (n)	Percentage (%)
Very Interested	217	60%
Moderately Interested	109	30%
Slightly Interested	29	8%
Not Interested	7	2%

Note: All data presented here are fully anonymized and aggregated to ensure participant privacy in accordance with GDPR requirements.
